# Peripheral blood amyloid-β involved in the pathogenesis of Alzheimer’s disease via impacting on peripheral innate immune cells

**DOI:** 10.1186/s12974-023-03003-5

**Published:** 2024-01-04

**Authors:** Mingchao Shi, Fengna Chu, Feiqi Zhu, Jie Zhu

**Affiliations:** 1 Neuroscience Center, Department of Neurology, The First Hospital of Jilin University, Changchun, China; 2 Department of Neurobiology, Care Sciences & Society, Division of Neurogeriatrcs, Karolinska Institute, Karolinska University Hospital Solna, Stockholm, Sweden; 3 Cognitive Impairment Ward of Neurology Department, The Third Affiliated Hospital of Shenzhen University Medical College, Shenzhen, China

**Keywords:** Alzheimer’s disease, Amyloid-β, Peripheral immune cells, Aging, Inflammation

## Abstract

A key pathological factor of Alzheimer’s disease (AD), the most prevalent form of age-related dementia in the world, is excessive β-amyloid protein (Aβ) in extracellular aggregation in the brain. And in the peripheral blood, a large amount of Aβ is derived from platelets. So far, the causality between the levels of peripheral blood Aβ and its aggregation in the brain, particularly the role of the peripheral blood Aβ in the pathology of AD, is still unclear. And the relation between the peripheral blood Aβ and tau tangles of brain, another crucial pathologic factor contributing to the pathogenesis of AD, is also ambiguous. More recently, the anti-Aβ monoclonal antibodies are approved for treatment of AD patients through declining the peripheral blood Aβ mechanism of action to enhance plasma and central nervous system (CNS) Aβ clearance, leading to a decrease Aβ burden in brain and improving cognitive function, which clearly indicates that the levels of the peripheral blood Aβ impacted on the Aβ burden in brain and involved in the pathogenesis of AD. In addition, the role of peripheral innate immune cells in AD remains mostly unknown and the results obtained were controversial. In the present review, we summarize recent studies on the roles of peripheral blood Aβ and the peripheral innate immune cells in the pathogenesis of AD. Finally, based on the published data and our own work, we believe that peripheral blood Aβ plays an important role in the development and progression of AD by impacting on the peripheral innate immune cells.

## Introduction

Alzheimer’s disease (AD) is the most common age-associated dementia with progressive loss of memory and cognitive functions [1]. It is predicted that by 2050, approximately 115 million people will be affected by AD [2]. Without question, AD has become a major public health dilemma and the largest health and social crisis in the world [3]. The neuropathological features of AD include amyloid-β (Aβ) plaques and neurofibrillary tangles formed by intracellular accumulation of hyperphosphorylated tau protein (p-Tau), neuroinflammation, as well as neuron and synapse loss, etc. [4, 5]. Aβ42 peptide is the main component of senile plaques in the brain of AD patients. All long major pathogenic hypotheses of AD focus on the Aβ cascade and p-Tau accumulation. For a long time in the past, all efforts in the treatment of AD targeting the pathogenic Aβ or tau have failed to demonstrate clinical efficiency unfortunately [6, 7], suggesting that the pathogenesis of AD should be quite complex and multifactorial [8].

Although the hypotheses of Aβ cascade and p-Tau have been challenged, there is growing evidence that the hypotheses of Aβ cascade play a key role in the pathogenesis of AD [9]. Neuroimaging studies showed that Aβ plaques begin to deposit in brain ten years or more before the onset of cognitive decline [10], which is consistent with the fact that AD pathologic change is a chronic long-term neurodegenerative process. Based on the Aβ cascade hypothesis, the clearance of brain Aβ plaques through different pathway should be able to treat AD and cease its progression, which promotes the development of innovative anti-Aβ drugs in order to lower Aβ production and prevent Aβ aggregation in brain.

Excitingly, in July 2023, the U.S. Food and Drug Administration (FDA) granted traditional approval to lecanemab, an anti-Aβ monoclonal antibody (mAb), according to a confirmatory clinical trial demonstrating clinical benefits for AD patients. In general, the anti-Aβ monoclonal antibodies (mAbs), such as lecanemab can enhance plasma and central nervous system (CNS) Aβ clearance, leading to a decline Aβ burden through the peripheral sink mechanism of action [11, 12], by which mAbs binding to Aβ in peripheral blood may alter the balance between circulation and brain Aβ levels, thereby enhancing outflow of soluble Aβ from the brain via low density lipoprotein receptor related protein 1 (LRP1) expressed on the blood brain barrier (BBB) [13, 14]. At the same time, binding of mabs to Aβ in peripheral blood also might reduce Aβ levels in the brain by lowering Aβ inflow into the brain through binding to the receptor for advanced glycation end products [14–16]. Lecanemab can selectively bind to large, soluble Aβ protofibrils that are the most neurotoxic and contribute to the pathogenesis of AD, and reduces Aβ plaques formed in brain, and slows down the rate of cognitive decline in early AD patients by 27% [17–19]. However, the mechanisms and pathways that the peripheral blood Aβ affects the progression of AD are not fully understood, which is an increasingly interesting topic that deserves in-depth research.

AD has been considered as a systemic disease that involves the peripheral and central immune responses. Dysregulation of immune response is also one of the pathological features of AD [20, 21]. In the past decade, emerging evidence has shown that the peripheral innate immunity has pathological effects on AD via the alterations of the function and quantity of peripheral immune cells in the blood and promotion of these cells infiltrating into the brain, exacerbating neuropathy in AD [21–23]. Notwithstanding, the precise effects of the peripheral immune system on AD are still unclear and controversial, and the studies to explore the role of peripheral immune cells in AD brain are still at a relatively nascent stage. So far there is lack of sufficient study on the correlations between peripheral immunity and AD, especially, how the peripheral blood Aβ impacts on brain of AD.

In the present article, we primarily review the role of the peripheral blood Aβ in AD patients and its animal models, and the role of the peripheral innate immune cells in AD pathogenesis. Finally, we focus on exploring the mechanism behind whether the peripheral blood Aβ contributes to AD through impacting on the peripheral innate immune cell functions.

## Peripheral blood Aβ source

In human peripheral blood, platelets are the primary source of Aβ peptides and a large amount (more than 90%) of Aβ is derived from circulating platelets. Thus, platelets are direct contributors to the amount of peripheral blood Aβ. Among them Aβ40 peptide is considered predominant form in some reported studies [24–27]. The level of platelet APP isoforms is comparable to it in the brain [28]. It has been revealed that Aβ metabolism is associated with platelet [29]. More than 90% of blood Aβ40 and over 97% of blood Aβ42 are related to plasma lipoproteins in human [30]. In mice, the amount of Aβ in platelets is increased with age [31].

In addition, the liver is another origin of brain Aβ deposits and is involved in peripheral clearance of circulating Aβ in the blood [32], therefore, the liver can affect indirectly the peripheral blood Aβ levels. Recent study reported that the liver was the earliest affected organ in AβPP/PS1 mice, the animal model of AD. During Aβ pathology progression [33], the liver was the major organ responsible for plasma clearance of Aβ_40/42_ [34, 35]. In our recent study, we found that the liver weights of APP/PS-1 transgenic (Tg) mice, the animal model for AD, decreased significantly, and liver cell necrosis as well as lymphocyte infiltration increased obviously compared to wild type mice [36], which indicates that the liver plays important roles in the pathogenesis of AD through affecting on Aβ metabolism [32, 37, 38]. Other peripheral sources of Aβ are endothelial vascular cells [39] and skeletal muscle [40, 41], but they are not the main source of the peripheral blood Aβ.

Aβ deposition in the brain may potentially correlate to the level in the peripheral circulatory system [42]. The discrepancy of Aβ isoforms levels in blood may mirror alterations in brain efflux of Aβ from cerebrospinal fluid (CSF) to blood [43]. However, some studies indicated that blood Aβ mainly presents changes in peripheral synthesis, secretion and metabolism of Aβ as an apoprotein of lipoproteins [44–46]. Deane et al. demonstrated that Aβ peptides are able to proactively transit across the BBB [47, 48]. When the BBB was damaged, the exchange of Aβ peptides between brain and peripheral blood/tissues was increased clearly. Reduction of the peripheral blood Aβ is enough to decline Aβ levels in brain, and reconfirming peripheral blood Aβ does not come from the brain [49].

## Role of peripheral Aβ in the pathogenesis of AD

Circulating activated platelets can produce Aβ and proinflammatory mediators. Both of them amplify peripheral inflammation and endothelial senescence, leading to change the permeability of the BBB and contributing to Aβ across BBB into brain, accelerating Aβ deposition in the brain and enhancing Aβ level in peripheral blood [50, 51], which leads to a decline in learning and memory. All human platelets can produce a large amount of Aβ in the peripheral blood, but not everyone will develop AD, which should involve other factors and is worth further exploring.

A retrospective cohort study reported that aspirin, a traditional antipyretic analgesic, applied to prevent and treat ischemic heart disease and cerebral thrombosis, can inhibit platelet aggregation in vitro and activation in vivo. Thus aspirin may lower the risk of AD [52, 53], since inhibition of platelet aggregation can decrease Aβ release [54]. The underlying mechanisms may be aspirin inhibiting cyclooxygenase (COX) activity, improving synaptic dysfunction by impacting COX-dependent action [55, 56]. Human platelets can regulate Aβ release by the cyclic adenosine monophosphate (cAMP)/protein kinase A (PKA) pathway, by which enhanced cAMP can block the processing and secretion of Aβ [57].

Additionally, the mice received the platelets from aged APP/PS1 mice (AD mouse model) through the tail vein injection caused significantly learning and memory deficits and raised Aβ deposition when compared with the mice injected with plasma [31], suggesting that in peripheral blood, Aβ derived from platelets is able to enter into the brain through the BBB and deposits in the hippocampus, playing an crucial role in the pathogenesis of AD [58]. Unfortunately, so far the effect of aspirin on AD has been controversial and no clinical evidence was confirmed that aspirin was efficacious in reducing cognitive decline in patients [59]. However, aspirin can indeed diminish the levels of Aβ40, Aβ42 and tau in both platelets and plasma of mice, and lower Aβ40 level obviously in hippocampi of AD mice accompanied by a tendency to decline Aβ42 deposition [31].

Clearing brain-derived Aβ is through transporting to the periphery, while the liver is the largest organ responsible for the clearance of metabolites, including Aβ in the periphery probably. A systemic failure of cell-mediated Aβ clearance is a pivotal event in the pathogenesis of AD and contributes to AD occurrence and progression. More recently, Cheng et al. reported that approximately 13.9% of Aβ42 and 8.9% of Aβ40 were cleared from the blood when flowing through the liver. When Aβ receptor LRP-1 expression was down-regulated in hepatocytes of the aged animals, this clearing ability was declined. Aβ levels in both blood and brain interstitial fluid were increased when hepatic blood flow reduced significantly [34], which showed that physiologically, the liver can clear peripheral blood Aβ and regulate brain Aβ levels, suggesting that during aging, the decrease in Aβ clearance in the liver might be associated with the pathogenesis of AD [34]. In the livers of AD patients, Aβ degradation capacity decreased significantly due to the reducing expression of Aβ-degrading enzymes, including cathepsin D and insulin-degrading enzyme compared with normal people [45].

An increasing number of studies indicated that the early diagnosis of AD might be conducted via assessment of peripheral plasma contains biomarkers that predict the levels of Aβ in the brain [60, 61]. There are now well-validated blood biomarkers for Aβ and tau pathology, as well as neurodegeneration and astrocytic activation in AD, including Aβ40, Aβ42, glial fibrillary acidic protein (GFAP), neurofilament light chain (NfL) and P-tau181 as well as the ratio Aβ42/Aβ40 [60, 62, 63]. Young people’s blood or plasma transfused to elderly patients can effectively lighten AD symptoms [64]. Overall, the above studies clearly evidenced the association between the peripheral blood Aβ and Aβ level/deposit in brain of AD, and the peripheral blood Aβ is a crucial causative factor in AD involving in the occurrence and progression of AD definitely. Furthermore, Aβ deposition in the brain may potentially correlate to the level in the peripheral circulatory system [65]. Based on this fact, the BBB could be one possible way of communication between the brain and periphery [66]. It has been reported that the BBB permeability of AD patients is higher than that of non-AD patients, so the peripheral blood Aβ is more likely to impact the stable environment of the CNS, and lead to the pathology of AD, such as Aβ deposit, inflammation and neurotoxicity [67].

## Impacting of the peripheral Aβ on peripheral innate immune cells

To date, dissecting the role of immune cells in AD pathogenesis has been challenging, particularly for the peripheral innate immune cells due to the fact that the exact role of the immune system in AD is still unclear and controversial. Previously immune dysfunction in the CNS was considered as a cause of the pathogenesis and progression of AD, while accumulating results showed key contributions of the peripheral immune system in AD as well. A consensus reached was that aberrant immune response was a cardinal feature of AD; meanwhile, a large amount of evidence suggested that pathological changes occurred in the central and peripheral immune responses throughout the entire AD process [23]. However, immune process raises a notable question how communication between the peripheral and central compartments occurs? Here, we emphasize to clarify the effects of the peripheral Aβ on peripheral innate immune cells, including neutrophils, monocytes, macrophages and natural killer (NK) cells. Understanding the causative roles (protective or harmful) of peripheral innate immune cells impacted by peripheral Aβ in AD will hopefully instruct therapeutic avenues to target the immune system at different stages of AD.

### Effect on neutrophils

Neutrophils are formed by bone marrow precursors in the bone marrow and are the most abundant white blood cells in humans and in mice [68]. Neutrophils are crucial in curbing invasive pathogens, antibacterial and antiviral responses, tissue repair, and mediating inflammation have become evident by phagocytosis, the release of antimicrobial molecules through degranulation, and containment and killing of pathogens via release of nuclear DNA [69]. Compared with young people, the activity of blood neutrophils in the healthy elderly is declined [70]. Particularly, lowered neutrophil function in AD was even greater in the later stages of the disease. Nevertheless, more neutrophils were observed in the early stages of AD than in age-matched healthy controls [70].

More than a decade ago, Baik, et al. have observed the peripheral neutrophils infiltrating into the brains of both AD patients and its Tg models (5xFAD and 3xTg-AD mice), and existing in the areas with Aβ deposits [71, 72]. At that time, it was obscure what factors touched off the recruitment of blood neutrophils toward Aβ plaques in brain? Because Aβ was not possible to directly recruit neutrophils, therefore, it was obscure what factors touched off the recruitment of blood neutrophils toward Aβ plaques in brain at that time. Recently, it has been found that Aβ42 triggered the lymphocyte function-associated antigen-1 (LFA-1) integrin high-affinity state and rapid neutrophil adhesion to integrin ligands. LFA-1 integrin can regulate neutrophil extravasation into the brain in vivo [72]. Particularly, Aβ is a formylpeptide receptor 2 agonist and a potent chemoattractant for attracting the peripheral blood leukocytes, monocytes as well as other immune cells entry into the brain and activated these cells there [73]. The brain is vulnerable to the effects of reactive oxygen species (ROS), while neutrophils produce a large amount of ROS in AD brain. Therefore, neutrophils appear to be the driving force behind AD.

In periphery, inflammatory mediators released by neutrophils can further cause changes in peripheral neutrophils count. Also BBB allows neutrophils to enter the brain and promotes the accumulations of neutrophils and Aβ there [74, 75]. For example, in acute colitis, neutrophils accelerated the accumulation of Aβ in the brain of the mouse model of AD and Kaneko et al. considered that neutrophil targeted therapy in AD may be a new strategy [75]. Recent studies have again demonstrated that neutrophils contribute to inflammation and disease progression in AD [72, 76–79], indicating that these effects of neutrophils via Aβ chemical attraction were harmful in AD. However, recently Sas et al. found a subset of neutrophils contributing to neuronal survival in the CNS [80]. Therefore, neutrophils may play double roles in the pathogenesis of AD. Anyway, pathogenic infiltration of peripheral immune cells, including neutrophils into the brain exacerbates AD pathology definitely [23, 81, 82], in which process, Aβ plays a pioneering role in pathogenicity due to attracting peripheral immune cells. Specially, depletion of infiltrating neutrophils by anti-Ly 6G or anti–Gr-1 antibody or suppressing neutrophil trafficking via LFA-1 alleviated pathological changes in two mouse models of AD (5XFAD and 3xTg) mice, which improved memory in mice those have cognitive impaired already and diminished the amyloid burden [72]. Moreover, infiltrating neutrophils in brain can induce neurotoxicity by releasing IL-17 that is a cytotoxic cytokine of neurons and mediates the destruction BBB, neutrophil extracellular traps and myeloperoxidase [72].

Aβ as a potent chemoattractant attracts the peripheral blood immune cells entry into the brain through three possible routes: (1) BBB that has been evidenced breakdown and dysfunction in AD [83]; (2) meninges that are enable immune cells to bypass BBB entering the brain through special skull bone marrow channels [84]; (3) choroid plexus that acts as a portal for immune cells from bone marrow to enter the brain [85]. Therefore, peripheral immune cells can play a role in both healthy and diseased brains [86, 87]. Several studies showed that the ratio of neutrophil and lymphocyte in the blood was correlated with cognitive decline in AD [65, 88] and neutrophils are involved in the pathogenesis of AD at the early stages via mediating BBB damage, infiltration and intravascular adhesion of the CNS [72]. Hou et al. reported that the increase in neutrophil count and neutrophil lymphocyte ratio were related to a decrease in cognitive, memory and executive functions. At the same time, they found that raised neutrophil–lymphocyte ratio was associated with a lower level of Aβ and higher level of total tau (T-tau) of CSF, as well as the atrophy of the hippocampus [22]. The latest research further emphasizes that the high proportion of peripheral neutrophils to lymphocytes may reflect an imbalance between innate and adaptive immunity, and is related to greater Aβ deposition and longitudinal cognitive decline [89]. When compared with mild cognitive impairment (MCI) patients, there were higher proportion of harmful and highly reactive aging and immunosuppressive neutrophils in AD [23]. The high proportion of peripheral neutrophils to lymphocytes may reflect an imbalance between innate and adaptive immunity, and a larger proportion of Aβ deposition is related to a decrease in longitudinal cognitive ability [89].

In addition, there are significant changes in neutrophil homeostasis when compared the patients with slower decline cognitive function with the patients with faster decline cognitive function, indicating that the neutrophil immune phenotype reflects the stage of the disease, but also showed a decrease in cognitive ability [90]. The specific impact of peripheral Aβ on neutrophils is still unclear, but the pathway of the role of the peripheral Aβ in neutrophils revealed a complex network involving Aβ clearance [91].

### Effect on monocytes

Monocytes, an innate immune cell population, usually account for 3–8% of the total number of white blood cells in the blood. They are derived from bone marrow hematopoietic stem cells and eventually enter tissues and transform into macrophages. Monocytes are less often in the CNS. In the blood, they will float in the blood until they reach the site that requires an immune response, then they will transform into macrophages, clearing away harmful substances such as bacteria and viruses. The peripheral monocytes are heterogeneous cells that are divided into multiple subpopulations with diverse surface markers, heterogeneous transcriptional profiles, and diverse functions. Up to now, the study on the role of monocytes in AD and impacting of the peripheral Aβ on monocytes has been limited. More recently, Liu et al. reported that the ability of blood monocyte phagocytosis Aβ in AD declined and monocyte in AD mice showed decreased in energy metabolism accompanied by cellular senescence, aging related secretory phenotype and dysfunction of phagocytic function [92]. However, increasing blood monocyte Aβ phagocytosis by improving energy metabolism in vivo can reduce brain Aβ deposition and neuroinflammation, ultimately improving cognitive function [92]. Additionally, removal of Ly6C monocyte in APP/PS1 mice can induce an obvious increase of Aβ deposit in the cortex and hippocampus, uncovering the ability of Ly6C(lo) monocytes eliminating Aβ [93]. Town et al. found presence of crawling monocytes carrying Aβ in veins and their ability to circulate back into the bloodstream in AD mice observed by live intravital two-photon microscopy [93]. Although circulating monocyte can penetrate into the brain and clear Aβ in AD patients, when compared with monocyte in the healthy subjects, the effect of these monocytes on alleviating AD pathology was significantly poor, accompanied by limited phagocytosis and phenotype, and have been adjusted to an inflammatory state, which was in line with the study of AD mice [94]. In 2021, Better et al. uncovered that Aβ_42_-induced monocytes lowed IL-1β secretion in healthy elderly adults and MCI, but not effected on AD, moreover, Aβ_42_ stimulated monocytes of healthy older to produce IL-10 only, suggesting the impact of monocytes on AD pathology was very limited [23]. Transforming growth factor-β (TGF-β) signaling expressing on peripheral macrophages was blocked to lead to substantial infiltration and clearance of cerebral Aβ in the Tg2576 mice, the animal model for AD [93]. Monocytes infiltrating the brain of AD Tg mice (APP/PS1 and 5XFAD) reduced Aβ burden, and improved cognitive performance [95, 96], indicating a beneficial role of monocytes in AD pathology.

Furthermore, the lack of methyltransferase like 3 (METTL3) in monocytes derived macrophages improved Aβ caused cognitive function impairment in AD mice, which is due to METTL3 ablation attenuating the m6A modification in DNA methyltransferase 3A (Dnmt3a) mRNAs and consequently impairing YTH N6-methyladenosine RNA binding protein 1 (YTHDF1)-mediated translation of DNMT3A [97]. More recently, the emerging evidence found that angiotensin-converting enzyme (ACE) can degrade Aβ42 neurotoxic 42-residue long alloform, while monocytes overexpressing ACE has neuroprotective properties in AD [98]. In addition, the peripheral myeloid cells infiltrating into brain tissue alleviated Aβ deposition and improved cognitive function in AD mouse models [93, 99, 100]. But, using brain-resident myeloid cells did not alter Aβ deposition in two mouse models of AD (APP23 and APP/PS1 mice) [101, 102], indicating that peripheral monocytes and microglia play their respective roles in clearing Aβ.

The latest findings indicate the interaction between cytokine tissue inhibitor of metalloproteinases-1 (TIMP-1) that triggered glucose uptake and proinflammatory cytokine expression in human monocytes, and members of the amyloid precursor protein (APP) family, namely APP and amyloid precursor protein-2 (APLP2) proved by confocal microscopy. TIMP-1 expression positively correlated with monocyte activation and proinflammatory cytokine production in cancer patients [103]. However, the impact of TIMP-1 on AD monocytes activation and its potential molecular mechanisms are largely unclear, at least one point can be understood: in AD, TIMP-1 activation of human monocytes is related to inflammation caused by Aβ. Infiltration of AD monocytes into the CNS relied on C–C motif chemokine receptor 2 (CCR2) and blockade of CCR2 in AD mice (App_swe_/PS1 and Tg2576) enhanced Aβ pathology and deteriorated memory impairment [99, 100]. In the process of Aβ42 recruiting peripheral monocytes, the translocator protein-18 kDa (TSPO) that is a transmembrane protein and overexpressed in response to neuroinflammation, involved in modulating this process [104]. It has been found in 1999 that benzodiazepine-induced chemotaxis is impaired in monocytes from patients with generalized anxiety disorder [105], suggesting that future therapeutic interventions aim at modulating monocytes motility toward the CNS.

The study by live two-photon imaging observed that the patrolling monocytes climbed to Aβ+ on the lumen wall of vein, internalized Aβ and circulated back into the bloodstream, hinting that monocytes can clear vascular Aβ of AD [106]. In this year, Uekawa et al. reported that brain border-associated macrophages (BAM) through the Aβ-binding innate immunity receptor CD36 lead to cognitive impairment. The absence of CD36 in BAM also declines brain Aβ40 without affecting plaques in the brain, and enhanced the vascular clearance of exogenous Aβ [107]. It has been evidenced that macrophages of AD patients have interrelated defects in the transcriptome, glycome, Aβ phagocytosis, and Aβ degradation [108].

As the major innate immune cells, peripheral macrophages are the chief phagocytes in the periphery [109] and their role of infiltrating into the CNS and association with Aβ deposit have been contentious. Macrophages can attract other cells migrating to injured or infected tissues via releasing signal molecules [109]. Since the similarity in phenotype between the macrophages and microglia, it is difficult to fully differentially distinguish them [110, 111], thus, most studies on AD focus on either a single cell or a joint analysis of both. Wisniewski et al. analyzed these two types of cells using an electron microscopy and considered that macrophages were more effective to eliminate Aβ plaques than microglia [112]. In response to Aβ stimulation, macrophages tend to produce less inflammatory cytokines than microglia [113]. In year 2020, Reed-Geaghan et al. reported that Aβ plaque was surrounded by microglia rather than infiltrating macrophages [114], speculating that microglia are more impactful in clearing Aβ plaque than macrophages. A new subset of macrophages has been found within the CNS in the border region areas, including the pia mater, perivascular space and choroid plexus and are replaced by circulating monocytes [115–117].

In short, these data showed that the study on the role of monocytes themselves and the effect of Aβ on circulating monocytes in AD are limited and is still unclear. More studies are needed to determine the direct role of circulating monocytes in AD and impact of Aβ on circulating monocytes.

### Effect on NK cells

As innate immune cells, NK cells represent approximately 15% of peripheral blood lymphocytes and are a subpopulation of cytotoxic lymphocytes. NK cells are divided into several NK cell subsets according to the differential expression of some phenotypical and functional markers. NK cells have an immunomodulatory role and accompanied by high secretion of cytokines and chemokines without pre-stimulation, such as interferon-γ (IFN-γ) [118], and also can kill other cells. The increased ratio of cytotoxic NK cells in the periphery was considered as a preclinical sign of AD and one of the Aβ neuropathological mechanism [119].

Importantly, NK cells can also stimulate macrophages contributing to chronic inflammation in the CNS of AD [118, 120]. During the aging process, the cytotoxic activity of NK cells in healthy people is impaired obviously compared to young people [70]. However, the role of NK cells in AD has always been disputed. Araga et al. reported a lowered cytotoxic function of NK cells in AD patients compared to healthy subjects [121, 122]. But Solerte et al. obtained an opposite result, which showed significantly enhanced levels of TNF-α and IFN-γ, as well as higher cytotoxic capacity of NK cells in AD when compared to healthy elderly subjects [123, 124]. In mild AD patients, no change in NK cell activation capacity was observed through comparing the expression of CD107a, a marker for granular release, and levels of granzyme B and IFN-γ [125], whether NK cells are altered in severe AD patients is not clear. In Rag2^–/–^/Il2rγ^–/–^deficient mice crossed with 5xFAD mice, NK cells, T cells, and B cells do not develop accompanied by raising Aβ levels [126]. However, in Rag2–/– mice crossed with PSAPP mice without T and B cells with NK cell-sufficient, there was a reduce Aβ plaques [127], which implies that NK cells may be more involved in clearing Aβ plaques compared to T and B cells, however, this warrants further investigation in such mice.

Similar to neutrophils, NK cells can also infiltrate the brain of the APP/PS1 mice causing Aβ pathology [115, 128, 129], however, neutrophils not only infiltrate the brain of three mouse models of AD (APP/PS1, 5 × FAD and 3 × Tg-AD mice) [130, 131], but also infiltrate the brains of AD patients. Up to now, the research on the infiltration of NK cells into the AD brain is limited. Using single-cell RNA (scRNA) sequencing data of sorted NKs (from datasets GSE181279 and GSE142853) illustrated the landscape of immune cells and immunity-related genes characteristics from the peripheral blood mononuclear cells in AD to confirm NK infiltration in the AD brain, which showed that the peripheral NK cells may infiltrate the brain and contribute to neuroinflammation in AD patients [129]. However, further in vivo studies are required to validate the CNS infiltration of peripheral NK cells and to investigate their role in AD. Meanwhile, the studies proposed that signal transducer and activator of transcription 3 (STAT3) may play a critical role in NK activation and infiltration into the brain in AD [129]. Due to the fact that NK cells do not require prior activation to secrete cytokines and chemokines to play their role, we speculate that circulating NK cells do not need the peripheral Aβ stimulation directly. Whether NK cells infiltration is needed Aβ as a potent chemoattractant for attracting them entry into the brain, it remains unclear. However, it is certain, NK cells are early responders who can receive the signals from target organs and quickly coordinate local inflammation upon arrival and involved the pathological changes of AD.

**Fig. 1:**
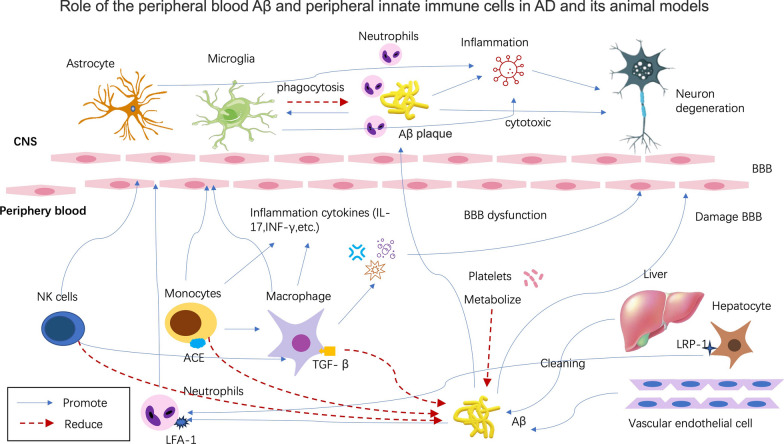
The role of the peripheral blood Aβ and innate immune cells in AD and its animal models. In human peripheral blood, a large amount of Aβ is derived from circulating platelets. And the liver is another origin of brain Aβ deposits and it is also a major organ responsible for cleaning up circulating Aβ in the blood. Clearing brain-derived Aβ is through transporting to the periphery. LRP-1 of Aβ receptor expression on hepatocytes of the aging animals can regulate Aβ clearance ability. Innate immune cells of the peripheral blood, including neutrophils, NK cells, monocytes and macrophages infiltrate into the CNS in AD and its animal models under Aβ chemotaxis, as well as produce inflammatory cytokines, such as IL-17 and IFN-γ. Aβ42 triggered LFA-1 regulates neutrophil extravasation into the brain. Meanwhile circulating activated platelets produce both Aβ and proinflammatory mediators, which could amplify peripheral inflammation and endothelial senescence, leading to change the permeability of the BBB and contributing to Aβ across BBB into brain, as well as accelerating Aβ deposition in the brain and enhancing Aβ level in peripheral blood. In the CNS, infiltrated peripheral innate immune cells and inflammatory mediators can stimulate astrocytes and microglia activation to produce inflammatory cytokines, which promote brain Aβ deposits and induce AD pathology. Finally, this leads to neuronal cell death, synaptic degradation and inflammation as well as gliosis, further exacerbating neurodegeneration and ultimate causing dementia

The role of the peripheral blood Aβ and peripheral innate immune cells in AD and its animal models is presented in Fig. 1.

## Conclusion

The peripheral blood Aβ is a potent chemoattractant for the peripheral innate immune cells infiltration into brain of AD, which is a crucial step to cause pathological changes in AD. There are communications between peripheral and the CNS in AD through several pathways, although the directionality and timing of these pathways are poorly understood.

In the early stage of disease, the peripheral Aβ is involved in the pathogenesis of AD through activating innate immune cells and promoting them to secretion of inflammatory cytokines and molecules leading to enhancing the BBB permeability or damage BBB. In the late stage, the peripheral Aβ may activate the peripheral and central inflammatory processes by affecting the proliferation and differentiation of innate immune cells. The recruitment of the peripheral innate immune cells may lead to increased production of proinflammatory cytokines by microglia, promoting the recruitment of more peripheral innate immune cells to move to Aβ plaques of brain. The peripheral innate immune cells could participate in engulfing and clearing the Aβ plaques in AD. Colocalization of innate immune cells with Aβ plaques is now a well-recognized neuropathological feature of AD. Based on the facts that (1) Aβ deposition in the brain may potentially correlate to the level in the peripheral circulatory system**,** and (2) anti-Aβ mabs therapy in AD is reducing blood Aβ levels through the peripheral sink mechanism of action, thus promotes CNS amyloid transforming to produce soluble monomers, across BBB to restore the decreased blood Aβ levels, we considered that the peripheral blood Aβ contributing to AD pathology is via impacting the peripheral innate immune cells partly. These give us a better understanding of the effects of the peripheral blood Aβ on the peripheral innate immune cells in AD pathology. However, due to insufficient study in this area, further investigation is necessary to understand the relationship between the peripheral Aβ and the peripheral innate immune cells in AD.

## Data Availability

Not applicable.
